# Integrating Bulk and Single-Cell RNA-Seq Data to Identify Prognostic Features Related to Activated Dendritic Cells in Clear-Cell Renal-Cell Carcinoma

**DOI:** 10.3390/ijms25179235

**Published:** 2024-08-26

**Authors:** Zijian Ye, Yifan Zhang, Jialiang Xu, Kun Li, Jianning Zhang, Deyana Ivanova, Xin Zhang, Siqi Liao, Liqi Duan, Fangfang Li, Xuemei Chen, Yingxiong Wang, Meijiao Wang, Biao Xie

**Affiliations:** 1 Department of Physiology, School of Basic Medical Science, Chongqing Medical University, Chongqing 400016, China; 2020220505@stu.cqmu.edu.cn (Z.Y.); 2020220523@stu.cqmu.edu.cn (Y.Z.); 2022110004@stu.cqmu.edu.cn (J.X.); 2020220130@stu.cqmu.edu.cn (K.L.); 2020220512@stu.cqmu.edu.cn (J.Z.); 2020221770@stu.cqmu.edu.cn (X.Z.); 2020220139@stu.cqmu.edu.cn (S.L.); 2020220391@stu.cqmu.edu.cn (L.D.); 2 Department of Biostatistics, School of Public Health, Chongqing Medical University, Chongqing 400016, China; 3 Department of Medicine, Division of Endocrinology, Diabetes and Hypertension, Brigham and Women’s Hospital, Harvard Medical School, Boston, MA 02115, USA; deyana.ivanova@yahoo.com; 4 Joint International Research Laboratory of Reproduction and Development of the Ministry of Education of China, School of Public Health and Management, Chongqing Medical University, Chongqing 400016, China; lifangfang715@126.com (F.L.); chenxuemei@cqmu.edu.cn (X.C.); yxwang@cqmu.edu.cn (Y.W.)

**Keywords:** dendritic cells, immunotherapy, molecular docking, immune activation, prognostic model

## Abstract

Dendritic cells (DCs) serve as key regulators in tumor immunity, with activated DCs potentiating antitumor responses through the secretion of pro-inflammatory cytokines and the expression of co-stimulatory molecules. Most current studies focus on the relationship between DC subgroups and clear-cell renal-cell carcinoma (ccRCC), but there is limited research on the connection between DCs and ccRCC from the perspective of immune activation. In this study, activated DC genes were identified in both bulk and single-cell RNA-seq data. A prognostic model related to activated DCs was constructed using univariate, multivariate Cox regression and LASSO regression. The prognostic model was validated in three external validation sets: GSE167573, ICGC, and E-MTAB-1980. The prognostic model consists of five genes, *PLCB2*, *XCR1*, *IFNG*, *HLA-DQB2*, and *SMIM24*. The expression of these genes was validated in tissue samples using qRT-PCR. Stratified analysis revealed that the prognostic model was able to better predict outcomes in advanced ccRCC patients. The risk scores were associated with tumor progression, tumor mutation burden, immune cell infiltration, and adverse outcomes of immunotherapy. Notably, there was a strong correlation between the expression of the five genes and the sensitivity to JQ1, a BET inhibitor. Molecular docking indicated high-affinity binding of the proteins encoded by these genes with JQ1. In conclusion, our study reveals the crucial role of activated DCs in ccRCC, offering new insights into predicting immune response, targeted therapy effectiveness, and prognosis for ccRCC patients.

## 1. Introduction

Renal-cell carcinoma (RCC) originating from the renal tubular epithelial cells includes multiple subtypes, such as clear-cell renal-cell carcinoma (ccRCC), chromophobe carcinoma, and papillary carcinoma [1,2]. RCC has a rising worldwide incidence rate at an estimated 400,000 new cases annually, with ccRCC being the predominant subtype, constituting approximately 70–80% of all RCC cases [3,4]. Common treatment modalities for ccRCC include partial or total nephrectomy, targeted therapy, immunotherapy, radiotherapy, and chemotherapy. Surgical resection is suitable only for early-stage ccRCC, while advanced-stage ccRCC often requires targeted therapy and immunotherapy [5,6]. Recently, the combination of immunotherapy and targeted therapy has been widely used in advanced-stage ccRCC patients [7,8]. Dendritic cells (DCs) are pivotal in immunotherapy. They recognize and present antigens from both endogenous and exogenous sources and activate T cell immune responses. Additionally, DCs can promote the formation of immune memory, leading to a persistent immune response against tumors and reducing the risk of recurrence [9]. In immunotherapy, DC vaccines loaded with tumor antigens are employed to stimulate an anti-tumor immune response [10,11]. Currently, numerous studies have examined DC subpopulations in tumor prognosis. However, there is an absence of DC-related prognostic models constructed from the perspective of immune activation to predict patient outcomes in ccRCC. This highlights the urgent need to establish a prognostic model associated with activated DCs.

DCs are pivotal “immune supervisors” in tumor immunity, participating in and regulating the immune response to tumors [12,13]. Activated DCs and inactivated DCs exhibit distinct differences in function and characteristics. Regarding immune activation status, activated DCs are typically in an immune-activated state, presenting antigens and activating T cells to promote a strong immune response. In contrast, inactivated DCs are in a resting or low-activity state, mainly maintaining immune balance and immune tolerance [14,15]. Regarding co-stimulatory molecule expression, activated DCs typically express high levels of co-stimulatory molecules, such as CD80 and CD86, on their surface. These molecules can bind to ligands on T cells, providing a second signal that promotes T cell activation and proliferation. Activated DCs may express lower levels of immune-inhibitory molecules such as PD-L1. In terms of cytokine secretion levels, activated DCs usually secrete various pro-inflammatory cytokines such as IL-12 and IFN-γ, which activate T cells and enhance immune responses. Inactivated DCs can secrete immune-suppressive cytokines, including IL-10 and TGF-β, that inhibit excessive immune responses and maintain immune tolerance [16,17,18]. Therefore, DCs in various states have distinct roles and significance in immune regulation therapy. Focusing on activated DCs to establish a prognostic model is of great significance in estimating immune infiltration and immunotherapy status in tumor patients.

In this research, activated DC genes were identified by jointly analyzing bulk transcriptome data and single-cell transcriptome data. A prognosis model related to activated DCs was established through univariate and multivariate Cox analysis and LASSO regression. The stability of the prognostic model was validated in three validation sets. The activated DC-related prognostic model showed significant correlations with the pathological stages and somatic mutations of ccRCC. The five genes included in the prognostic model collaboratively remodel the immune microenvironment, significantly influencing the effectiveness of immunotherapy and targeted treatments. Additionally, these genes possess binding sites for JQ1, which can alter the antigen-presenting capabilities of DCs, thereby influencing T cell activation and immune response. This suggests that they may serve as potential competitive binding targets of JQ1, thereby participating in the activation process of DCs.

## 2. Results

### 2.1. Workflow of the Study

**Figure 1:**
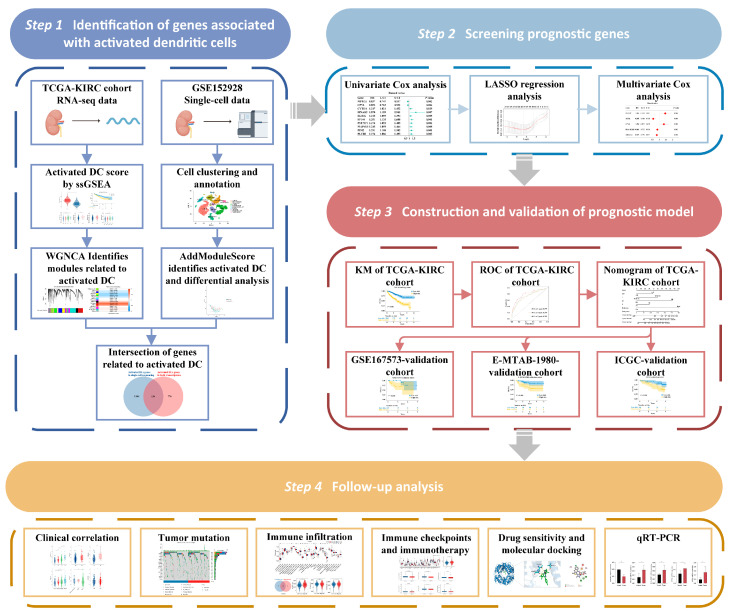
Flowchart of the study.

The main research approach of this article is divided into four parts, as depicted in Figure 1.

### 2.2. Activated DC Scores in Different Clinical Stages

**Figure 2:**
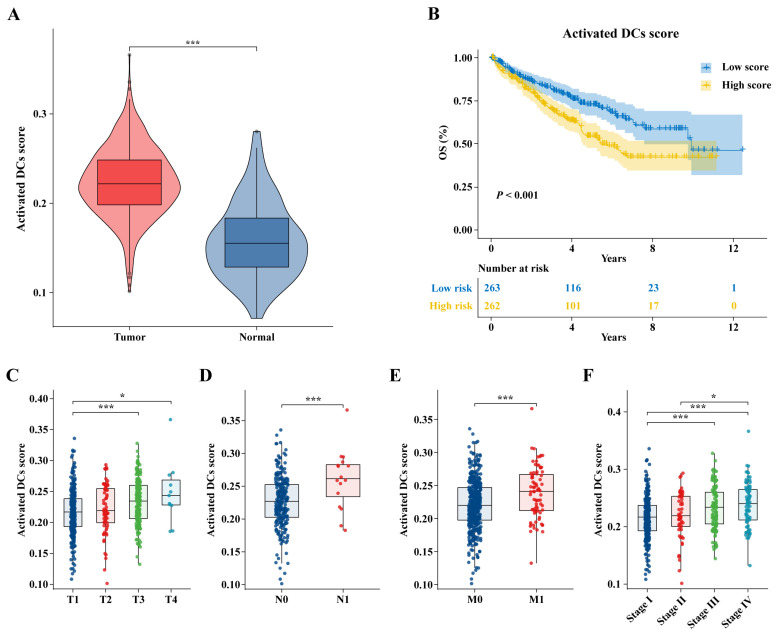
A high activated DC score correlates with poor prognosis of ccRCC in the TCGA-KIRC cohort. (**A**) Comparing the activated DC scores in ccRCC and adjacent normal tissues. (**B**) KM survival curve based on median activated DC scores. Comparison of activated DC scores between (**C**) T, (**D**) N, and (**E**) M stages and (**F**) pathological stages; * *p* < 0.05, *** *p* < 0.001.

The activated DC scores were calculated using the ssGSEA algorithm in the TCGA-KIRC cohort. The relationship between the activated DC scores and tumor development was investigated by comprehensively analyzing the differences in activated DC scores under different clinical conditions in the TCGA-KIRC cohort. The findings indicated the activated DC scores were markedly higher in tumors (*p* < 0.001, Figure 2A). The activated DC scores were significantly increased in T1 and T3 (*p* < 0.001, Figure 2C) and in T1 and T4 (*p* < 0.05, Figure 2C), demonstrating a strong positive correlation with the NM stage (*p* < 0.001, Figure 2D,E). In the pathological stage, the activated DC scores were significantly increased from pathological stage 1 to pathological stage 3 (*p* < 0.001, Figure 2F), from pathological stage 1 to pathological stage 4 (*p* < 0.001, Figure 2F), and from pathological stage 2 to pathological stage 4 (*p* < 0.05, Figure 2F). The Kaplan–Meier (KM) survival curve showed that patients with low activated DC scores had better overall survival (OS) (Figure 2B). These findings underscored the significant association between activated DC scores and the invasive, metastatic, and prognostic attributes of ccRCC patients.

### 2.3. WGCNA Identified Modules Associated With Activated DC Score

**Figure 3:**
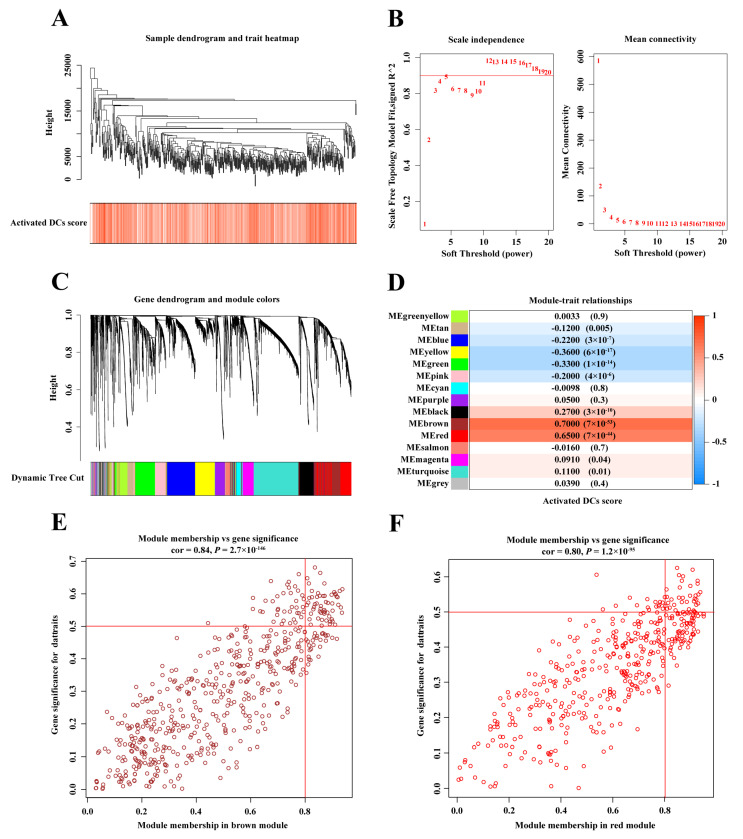
WGCNA identifies key modules associated with activated DCs in the TCGA-KIRC cohort. (**A**) Sample clustering and heatmaps showing activated DCs. (**B**) Selection of the soft threshold. (**C**) Gene clustering modules. (**D**) Heatmap showing the correlation between gene clustering modules and activated DC score phenotype. Gene significance is highly correlated with module membership in the (**E**) brown and (**F**) red modules.

ccRCC patients in the TCGA-KIRC cohort were clustered according to their activated DC score, and the sample clustering tree was plotted (Figure 3A). The results showed good clustering among samples without significant outlier samples. The optimal soft threshold was selected as four (Figure 3B), and a gene dendrogram based on gene correlations was constructed to form 15 gene modules, with each color representing a different module (Figure 3C). Subsequently, the correlation between each module and the activated DC scores was evaluated. The brown and red modules showed the strongest correlation with activated DC scores (Figure 3D), with correlation coefficients of gene significance and module membership reaching 0.84 and 0.80, respectively (Figure 3E,F). This indicates excellent construction quality for the brown and red modules. In total, 910 genes were identified as related to activated DCs from the two modules (Table S1).

GO analysis indicated that the genes in the brown module were primarily enriched in immune response activation processes, such as positive regulation of cytokine production, positive regulation of defense response, and immune response-regulating signaling pathways (Figure S1A). KEGG results indicated that the genes in the brown module were enriched in immune-related pathways, such as cytokine–cytokine receptor interactions and Staphylococcus aureus infection (Figure S1B). Genes in the red module were also similarly enriched in immune response regulation processes, such as leukocyte-mediated immunity, lymphocyte-mediated immunity, and regulation of T cell activation (Figure S1C). KEGG analysis revealed that genes in the red module were also enriched in immune-related pathways, such as cytokine–cytokine receptor interactions, cell adhesion molecules, and antigen processing and presentation (Figure S1D). These results confirmed the association of genes in the red and brown modules with activated DCs.

### 2.4. Identification of Activated DC Genes in Single-Cell Transcriptome Data

**Figure 4:**
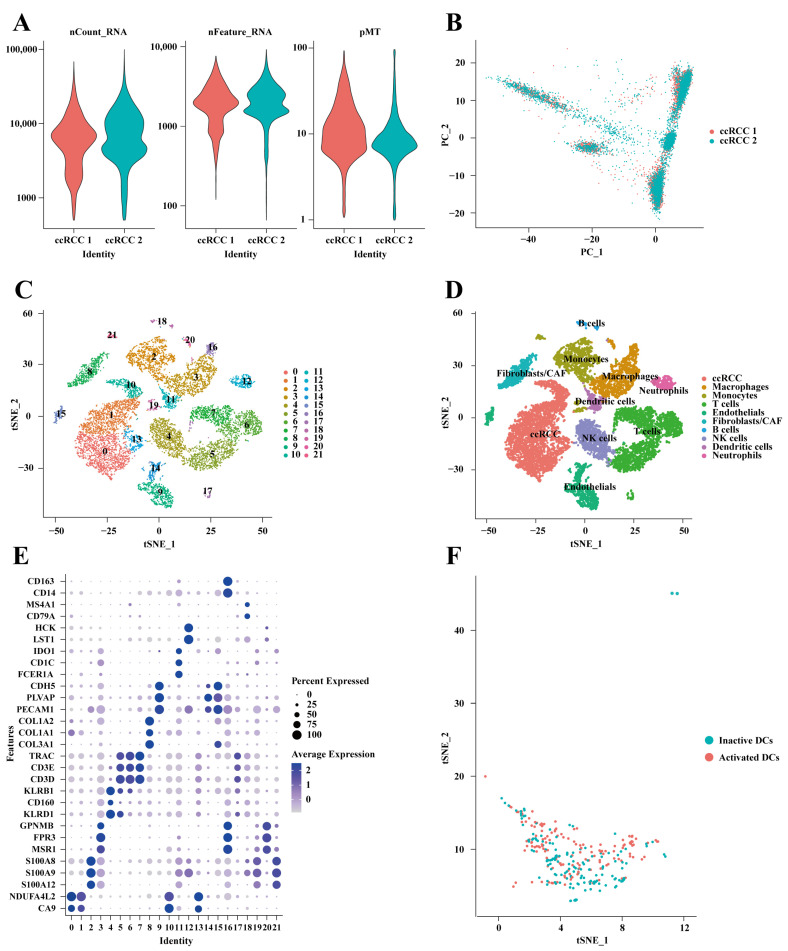
Identification of genes associated with activated DCs at the single-cell transcriptome level. (**A**) nFeatures, nCount, and pMT for quality control in single-cell datasets. (**B**) Principal component analysis for dimensionality reduction. (**C**) tSNE analysis revealed 21 cell clusters. (**D**) Annotated cell clusters. (**E**) The expression levels of characteristic genes. (**F**) Distribution of activated DC scores within the DCs.

Prior to the quality control analysis of the GSE152938 single-cell dataset, the number of genes with sequencing depth and the percentage of mitochondrial genes were analyzed (Figure 4A). After quality control using the above criteria, 12,915 core cells were retained for further analysis. Subsequently, PCA dimensionality reduction was performed (Figure 4B). Core cells were classified into 21 independent cell clusters using the t-SNE algorithm (Figure 4C). Combined with the CellMarker database and the reference literature, marker genes were identified for different clusters, and the expression levels were visualized using a bubble plot (Figure 4E). Finally, 10 cell clusters were obtained, including B cells, macrophages, tumor cells, T cells, monocytes, endothelial cells, fibroblasts, NK cells, DCs, and neutrophils (Figure 4D). Then, the DC clusters were isolated, and the ‘AddModuleScore’ function was used to evaluate the enrichment score of the activated DC gene set from the TISIDB database. Cells were divided into activated DCs and inactivated DCs based on the median enrichment scores, with cells scoring above the median considered as activated DCs and cells with scores below the median considered as inactivated DCs (Figure 4F). Subsequently, differential expression analysis was performed between activated and inactivated DCs, identifying differentially expressed genes (DEGs) as activated DC genes (Table S2).

GO revealed that activated DC genes were enriched in immune response regulation processes, such as activation of the immune response and positive regulation of leukocyte activation (Figure S1E). KEGG analysis indicated that activated DC genes were associated with immune-related pathways, such as human papillomavirus infection, primary immunodeficiency, and Fc gamma R-mediated phagocytosis (Figure S1F).

### 2.5. Construction and Validation of the Activated DC-Related Prognostic Model

**Figure 5:**
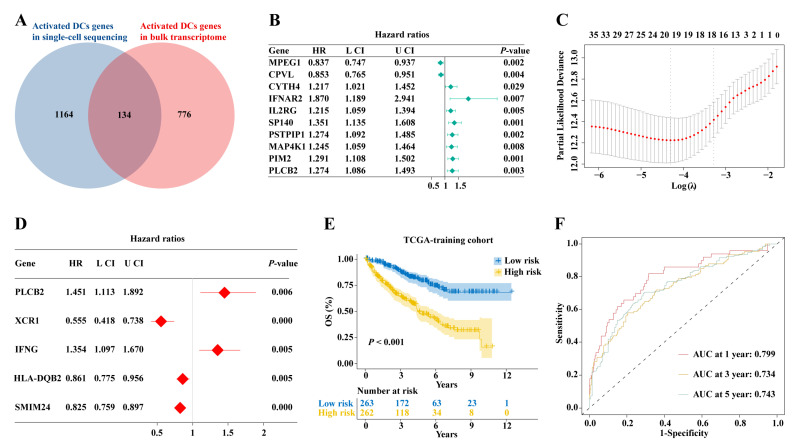
Constructing a prognostic model of activated DCs. (**A**) Identifying common activated DC-related genes in bulk and single-cell sequencing. (**B**) Univariate Cox analysis. (**C**) LASSO regression analysis. (**D**) Multivariate Cox analysis confirmed five genes with which to construct the prognostic model. (**E**) The KM survival curve for the TCGA-KIRC cohort. (**F**) Time–ROC curves.

**Figure 6:**
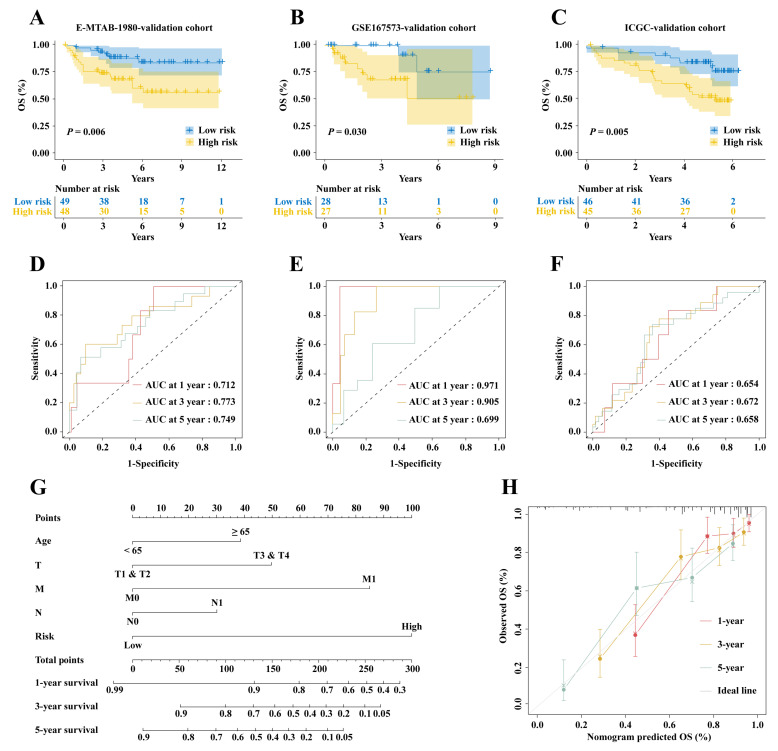
Validate the prognostic model of activated DCs and construct a nomogram. KM survival curves of (**A**) the E-MTAB-1980 cohort, (**B**) the GSE167573 cohort, and (**C**) the ICGC cohort. Time-dependent ROC curves of (**D**) the E-MTAB-1980 cohort, (**E**) the GSE167573 cohort, and (**F**) the ICGC cohort. (**G**) Nomogram based on clinical features and risk scores. (**H**) Calibration curves.

The intersection of activated DC genes from bulk transcriptome and single-cell data yielded 134 genes (Figure 5A). Univariate Cox analysis was initially used to screen genes associated with ccRCC patient prognosis, resulting in 40 significantly associated genes (*p* < 0.05), with the top 10 plotted in a forest plot (Figure 5B). Subsequently, LASSO regression analysis was performed, selecting λ.1se as the threshold, resulting in 18 genes. Finally, multivariate Cox analysis identified five genes (*PLCB2*, *XCR1*, *IFNG*, *HLA-DQB2*, and *SMIM24*), which were used to develop the prognostic model (Figure 5C). The risk score calculation method for this model is as follows: risk scores = (0.372 × *PLCB2* expression) + (−0.589 × *XCR1* expression) + (0.303 × *IFNG* expression) + (0.15 × *HLA-DQB2* expression) + (−0.192 × *SMIM24* expression). The KM survival curve demonstrated a markedly worse OS for high-risk patients (*p* < 0.001, Figure 5E). The AUC values for 1-, 3-, and 5-year predictions were 0.799, 0.734, and 0.743 (Figure 5F). Validation was conducted in the E-MTAB-1980 cohort (Figure 6A), the GSE167573 cohort (Figure 6B), and the ICGC cohort (Figure 6C). The KM survival curves showed similar results to the training set, with AUC values of 0.712, 0.773, and 0.749 in the E-MTAB-1980 cohort (Figure 6D), 0.971, 0.905, and 0.699 in the GSE167573 cohort (Figure 6E), and 0.654, 0.672, and 0.658 in the ICGC cohort (Figure 6F).

Furthermore, clinical factors such as T, N, M, and age were combined to create a nomogram for predicting patient survival rates to aid clinical decision-making (Figure 6G). The calibration curve reflected the relationship between actual survival rates and the predicted survival rates from the nomogram, demonstrating their consistency.

### 2.6. Clinical Correlation Analysis in the TCGA-KIRC Cohort

**Figure 7:**
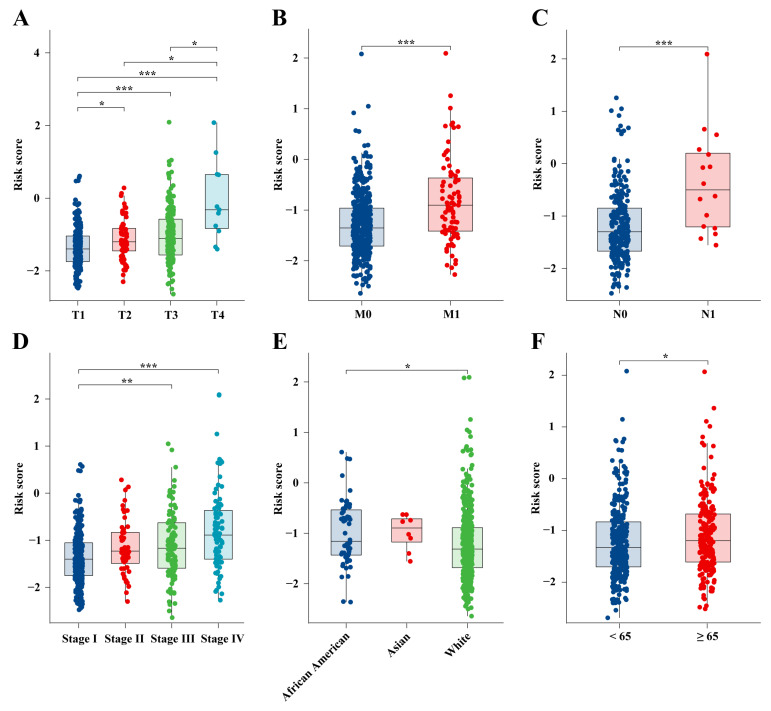
Differences in risk scores and clinical features in the TCGA-KIRC cohort. The differences in risk scores between the (**A**) T, (**B**) M, and (**C**) N stages, (**D**) the pathological stage, (**E**) ethnicity, and (**F**) age; * *p* < 0.05, ** *p* < 0.01, *** *p* < 0.001.

The risk scores calculated by the prognostic model were combined with the clinical staging of the ccRCC patients in the TCGA-KIRC cohort. Risk scores increased with the advancement in primary tumor size (Figure 7A). Among patients with distant metastasis, risk scores were significantly elevated (*p* < 0.001, Figure 7B), and in patients with lymph node metastasis, the risk scores also markedly increased (*p* < 0.001, Figure 7C). Similar trends were observed across clinical pathological stages, with risk scores rising as the stage progressed (Figure 7D). These results showed a strong correlation between the prognostic model and the growth, invasion, and metastasis of ccRCC tumors. Interestingly, when categorized by ethnicity, white patients exhibited significantly lower risk scores compared to African American patients (*p* < 0.05, Figure 7E). Among age groups, patients over 65 exhibited elevated risk scores (*p* < 0.05, Figure 7F).

### 2.7. Survival Analysis of Clinical Subgroups in the TCGA-KIRC Cohort

**Figure 8:**
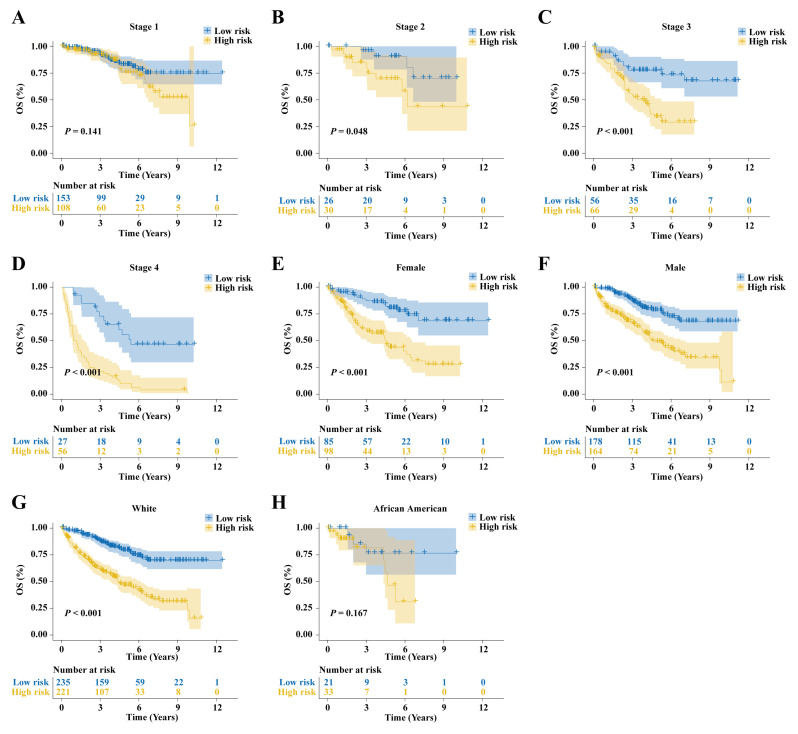
Stratified survival analysis in the TCGA-KIRC cohort. Prediction efficacy of the prognostic model in (**A**) pathological stage 1 (**B**) 2, (**C**) 3, (**D**) and 4, (**E**) female, (**F**) male, (**G**) white, and (**H**) African American populations.

Different ccRCC subgroups in the TCGA-KIRC cohort were divided according to pathological stage, gender, and ethnicity. The KM survival curve results showed significant differences, except for pathological stage 1 (Figure 8A) and the African American subtype (Figure 8H), where survival analysis was not meaningful. In all other subtypes, the low-risk group exhibited better OS with statistically significant differences. Notably, as the pathological stage progresses (Figure 8A–D), the KM survival curves indicate an increasing distinction in the prognostic model’s discriminatory ability. This suggests that the prognostic model offers superior predictive performance for patients in advanced clinical pathological stages.

### 2.8. Tumor Mutation Analysis in the TCGA-KIRC Cohort

**Figure 9:**
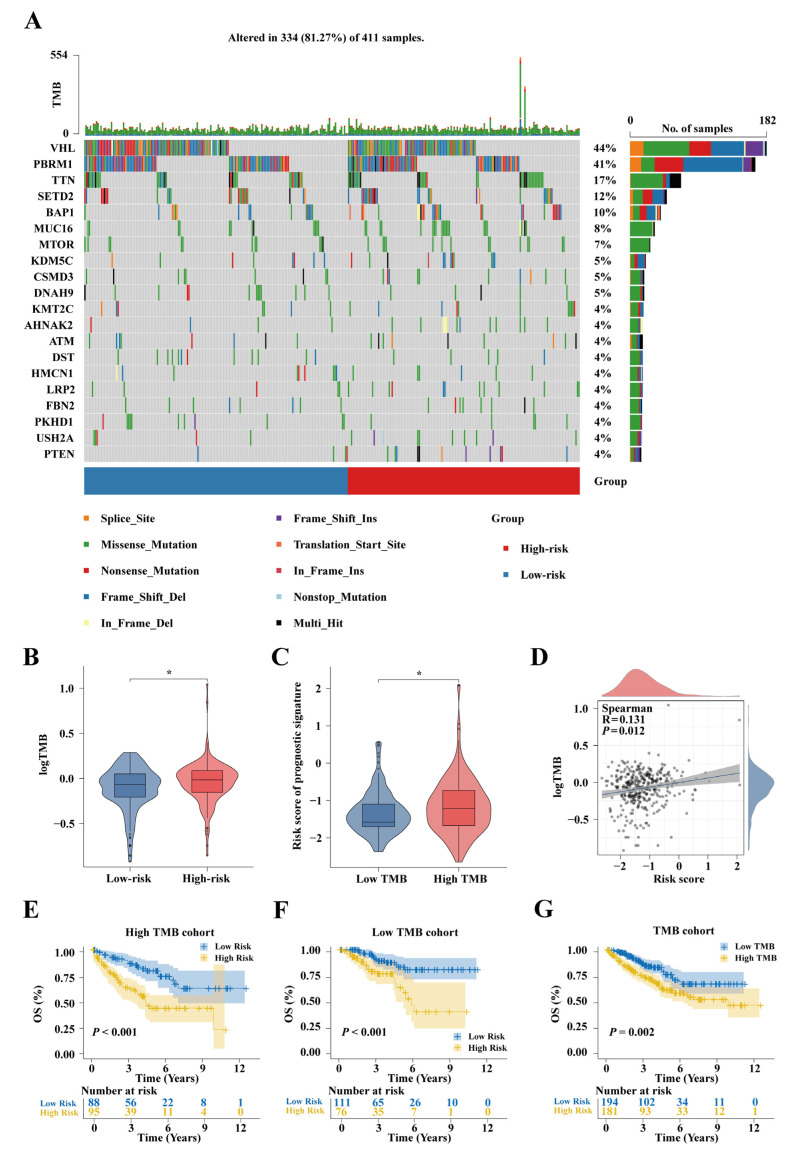
Somatic mutation analysis in the TCGA-KIRC cohort. (**A**) The top 20 genes with the highest mutation frequency. (**B**) Differences in TMB between the high- and low-risk groups. (**C**) Differences in risk scores between the high- and low-TMB groups. (**D**) Correlation analysis between TMB and risk scores. KM survival curves of the prognosis model in (**E**) high- and (**F**) low-TMB subtypes. (**G**) KM survival curves based on low- and high-TMB groups; * *p* < 0.05.

Waterfall plots of gene mutations in the TCGA-KIRC cohort showed that mutations in VHL and PBRM1 were more frequent in the low-risk group (Figure 9A). Assessment of Tumor Mutational Burden (TMB) revealed that high-risk patients exhibited higher TMBs (*p* < 0.05, Figure 9B). High-TMB patients had higher risk scores (*p* < 0.05, Figure 9C). A correlation analysis between risk scores and TMB revealed a significant positive correlation (*p* < 0.05, Figure 9D). KM survival curve analysis indicated that the low-TMB group had better OS (*p* = 0.05, Figure 9G). Subsequently, based on our developed prognostic model, we performed survival analysis on patients with high and low TMBs. The results indicated that low-risk patients showed superior OS in both the high-TMB (*p* < 0.001, Figure 9E) and low-TMB subgroups (*p* < 0.001, Figure 9F).

### 2.9. Immune Cell Infiltration in the TCGA-KIRC Cohort

**Figure 10:**
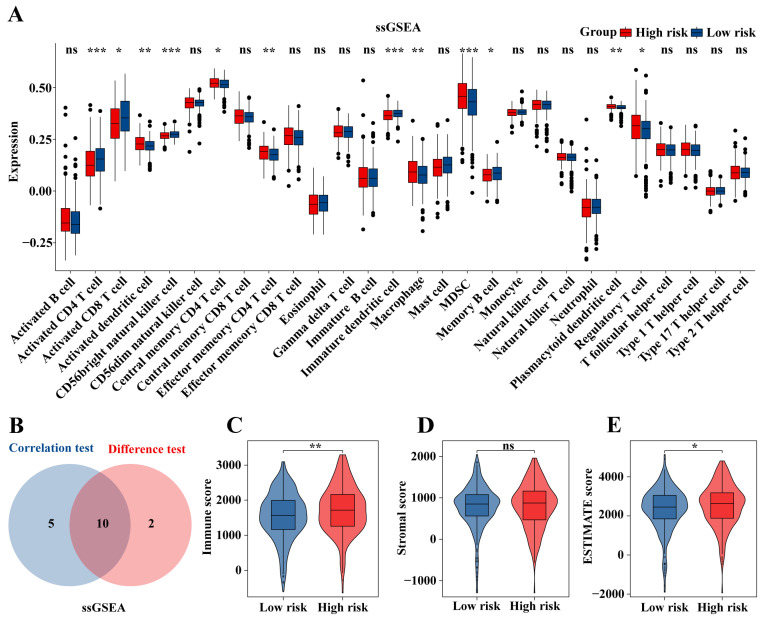
Immune cell infiltration in the TCGA-KIRC cohort. (**A**) Analysis of 28 immune cell types using ssGSEA algorithms. (**B**) Immune cells with statistically significant differences and correlations. Analysis of (**C**) immune scores, (**D**) stromal scores, and (**E**) ESTIMATE scores; * *p* < 0.05, ** *p* < 0.01, *** *p* < 0.001, ns, not significant.

The ssGSEA algorithm was used to assess immune infiltration in the TCGA-KIRC cohort. Immature DCs, activated CD4^+^ T cells, activated CD8^+^ T cells, and memory B cells had higher infiltration levels in the low-risk group. In contrast, activated DCs, CD56bright natural killer cells, central memory CD4^+^ T cells, effector memory CD4^+^ T cells, macrophages, MDSCs, Tregs, and neutrophils had higher infiltration levels in the high-risk group (Figure 10A). Correlation analysis was performed between risk scores and immune cell infiltration, and 15 types of immune cells were found to have significant statistical relevance (*p* < 0.05, Figure S2). The intersection of immune cells showing statistically significant results in both correlation and differential analyses yielded a total of 10 immune cells (Figure 10B). Notably, activated DCs were significant in both analyses. Additionally, the ESTIMATE algorithm was employed to assess the tumor microenvironment. The immune score (*p* < 0.01, Figure 10C) and the ESTIMATE score (*p* < 0.05, Figure 10E) were significantly elevated in the high-risk group, whereas the stromal score showed no significant difference (Figure 10D).

### 2.10. Predicting Immunotherapy and Targeted Therapy Responses in the TCGA-KIRC Cohort

**Figure 11:**
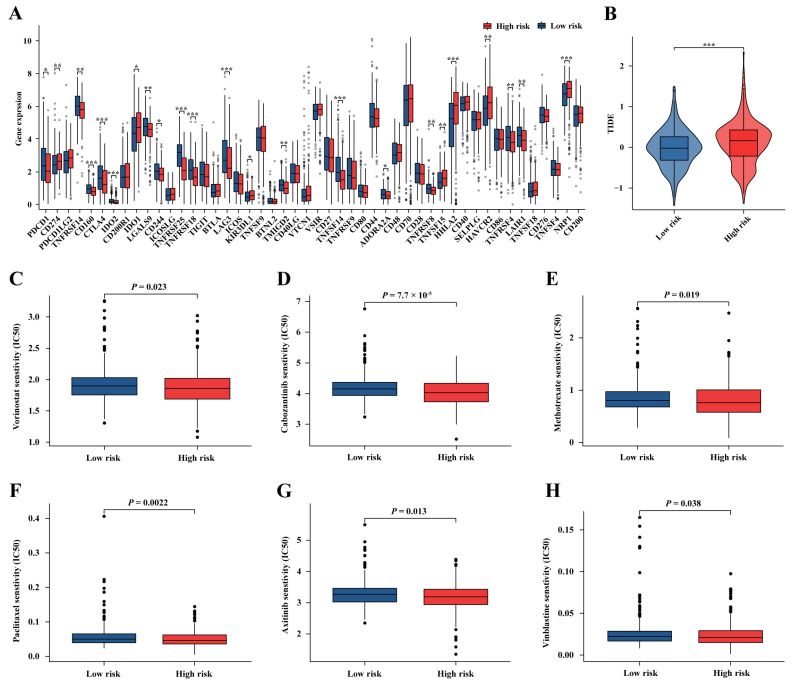
Predicting immunotherapy, chemotherapy, and targeted therapy responses in the TCGA-KIRC cohort. Differences in immune checkpoint expression (**A**) and TIDE score (**B**) between high- and low-risk groups. Differences in IC50 of (**C**) Vorinostat, (**D**) Cabozantinib, (**E**) Methotrexate, (**F**) Paclitaxel, (**G**) Axitinib, and (**H**) Vinblastine between high- and low-risk groups; * *p* < 0.05, ** *p* < 0.01, *** *p* < 0.001.

Immune checkpoint analysis revealed that the expression of most immune checkpoints was upregulated in the low-risk group (Figure 11A). Additionally, the TIDE scores were lower in the low-risk group, suggesting a higher sensitivity of the low-risk group to immune therapy (*p* < 0.001, Figure 11B).

To further investigate drug sensitivity in different risk groups, the IC50 of 16 commonly used ccRCC treatment drugs was analyzed. The results showed that Cabozantinib, Axitinib, Paclitaxel, Vinblastine, Vorinostat, and Methotrexate had lower IC50 values in the high-risk group, indicating better treatment efficacy (Figure 11C−H).

### 2.11. Drug Sensitivity Analysis and Molecular Docking

**Figure 12:**
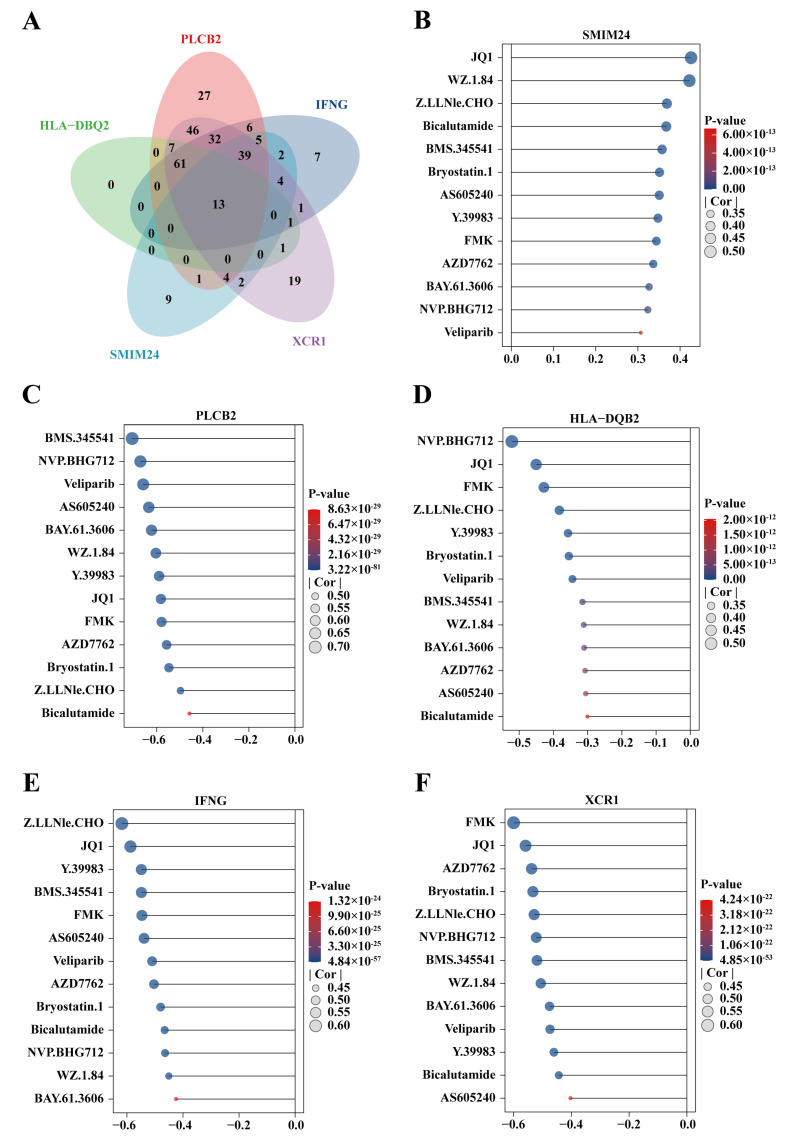
Drug sensitivity prediction for five activated DC-related genes. (**A**) Intersection of drugs showing a strong correlation between expression of five activated DC-related genes and predicted IC50. A lollipop chart displaying the correlation between the predicted IC50 of 13 drugs in the intersection and the expression of (**B**) HLA-DQB2, (**C**) SMIM24, (**D**) XCR1, (**E**) IFNG, and (**F**) PLCB2.

**Figure 13:**
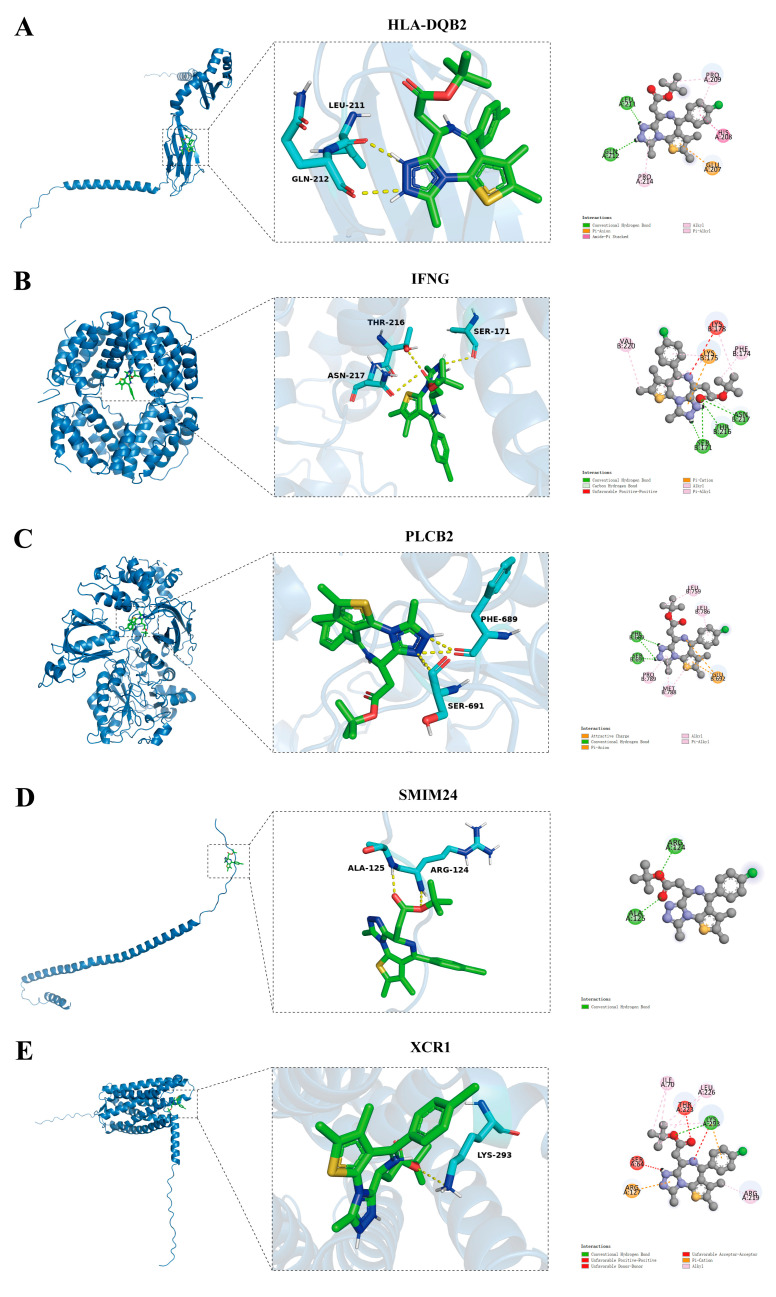
Molecular docking analysis. Prediction of binding sites between proteins encoded by (**A**) HLA-DQB2, (**B**) XCR1, (**C**) SMIM24, (**D**) IFNG, and (**E**) PLCB2 and JQ-1, where blue represents amino acid residues, green represents JQ-1, and yellow represents hydrogen bonds.

To explore potential drugs for each gene, a correlation analysis was conducted between the expression of five activated DC genes and the IC50 of 198 drugs. A threshold of correlation |cor| > 0.3 with *p* < 0.05 was used to select potential drugs for each gene. The intersection of potential drugs selected for the five activated DC genes was obtained (Figure 12A), resulting in 13 potential drugs. A lollipop plot was drawn for correlation analysis, showing that the expression of *SMIM24* was positively correlated with IC50 (Figure 12C), while the expression of *HLA-DQB2* (Figure 12B), *XCR1* (Figure 12D), *IFNG* (Figure 12E), and *PLCB2* (Figure 12F) genes was negatively correlated with IC50. Of note, JQ1 showed high correlation coefficients in the correlation analysis, so we chose JQ1 as the ligand and conducted molecular docking with the proteins encoded by *HLA-DQB2*, *XCR1*, *SMIM24*, *IFNG*, and *PLCB2*. JQ1 formed three hydrogen bonds with THR-216, SER-171, and ASN-227 of IFNG (Figure 13B), two hydrogen bonds with PHE-689 and SER-691 of PLCB2 (Figure 13C), two hydrogen bonds with ALA-125 and ARG-124 of SMIM24 (Figure 13D), and one hydrogen bond with LYS-293 of XCR1 (Figure 13E). Importantly, the binding energies were all below −5 kcal/mol, indicating strong binding affinity. Detailed binding energies are provided in Table 1.

### 2.12. Validation of the Expression of Five Activated DC-Related Genes

**Figure 14:**
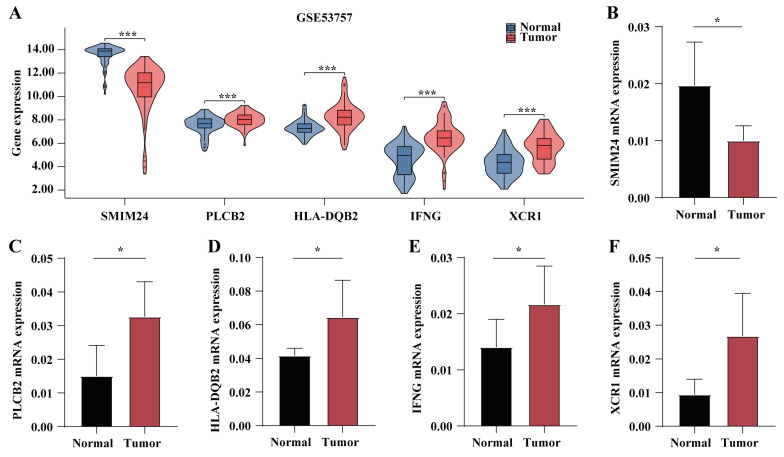
Validation of the expression of five activated DC genes. (**A**) The expression of these five activated DC-related genes was analyzed in GSE53757. qRT-PCR validated the expression of (**B**) SMIM24, (**C**) PLCB2, (**D**) HLA-DQB2, (**E**) IFNG, and (**F**) XCR1 in 15 paired tumor and adjacent non-tumor tissue samples obtained from the Department of Urology at the First Affiliated Hospital of Chongqing Medical University; * *p* < 0.05, *** *p* < 0.001.

GSE5375 was used for external validation of the expression of five activated DC genes. The results showed that, with the exception of *SMIM24*, which was highly expressed in normal tissues, *PLCB2*, *HLA-DQB2*, *IFNG*, and *XCR1* were all highly expressed in tumor tissues (Figure 14A). Additionally, qRT-PCR analysis of 15 paired tumor and adjacent non-tumor tissue samples, obtained from the Department of Urology at the First Affiliated Hospital of Chongqing Medical University, also showed consistent results, consistent with our bioinformatics predictions (Figure 14B–F).

## 3. Discussion

DCs are involved in tumor immune antigen presentation and immune activation. They have also been utilized to develop DC vaccines and DC transfer immunotherapy to enhance the patient’s immune response against tumors [19]. Stimulating ex vivo DCs to improve their antigen uptake and presentation capabilities has been a cornerstone of DC-based cancer therapies. Currently, research on DC biomarkers has predominantly focused on single-omics levels and individual biomarkers [20,21,22]. Zhang et al. [23] employed a similar approach to identify prognostic markers for DCs in lung adenocarcinoma. They collected single-cell sequencing data from five lung adenocarcinoma patients, performed clustering and annotation to identify DCs, and then directly searched for marker genes of DCs for subsequent analysis. In contrast to their study, our research not only identifies activated DC genes at the single-cell transcriptome level but also at the bulk transcriptome level, enhancing the accuracy of the identification. Shi et al. [24] focused on the prognostic markers of DC subtypes, including plasmacytoid DCs, conventional DCs, and tolerogenic DCs, in esophageal squamous cell carcinoma. Their analysis comprehensively revealed the roles of different DC subtypes in esophageal squamous cell carcinoma and their impact on patient prognosis. Compared to their study, we aim to explore DC subtypes and their prognostic markers from an immune activation perspective, examining their impact on the prognosis of ccRCC patients. Zhao et al. [25] utilized a combination of single-cell data and bulk transcriptomic data to investigate DC-related prognostic markers in lung adenocarcinoma, identifying CD1C as a predictor of patient prognosis and immune response. In contrast to their study, our research demonstrates stronger predictive capability by establishing a prognostic model that includes multiple genes associated with patient outcomes. Additionally, Zhao et al.‘s study lacks external dataset validation, whereas our findings have been thoroughly validated across three independent cohorts. Currently, there are no studies on DC-related prognostic markers in ccRCC, highlighting the novelty of our research. In this study, we integrated bulk transcriptomics and single-cell transcriptomics data for the first time to develop a prognostic model related to activated DCs, utilizing multiple biomarkers. This approach aims to improve the accuracy and value of prognostic assessments, predict patient outcomes and responses to immunotherapy, and identify potential therapeutic targets. Importantly, while previous studies on ccRCC prognostic models typically involved one or two external validation cohorts or relied on internal validation, our study utilized three external validation cohorts, thereby demonstrating the model’s robust predictive performance and stability.

The heterogeneity of immune cells may shape distinct tumor microenvironments. On one hand, CD8^+^ T cells, NK cells, and M1 macrophages have the ability to recognize and eliminate tumor cells, thus inhibiting tumor progression [26,27]. On the other hand, M2 macrophages, Treg cells, and MDSCs may display immune-suppressive characteristics, thereby promoting tumor growth, invasion, and metastasis [28,29,30]. We found that the immune microenvironment of high-risk ccRCC patients contains a higher number of immunosuppressive cells, such as Treg cells. Treg cells may suppress the activity of CD4^+^ and CD8^+^ T cells, thereby promoting immune tolerance. Our study revealed that activated DC scores were positively correlated with TNM and pathological stage progression, indicating enhanced tumor progression, metastasis, and invasion abilities. This indicates that patients with high activated DC scores are likely to be at an advanced stage. Additionally, clinical correlation analysis also demonstrated that advanced-stage patients have higher risk scores. This explains why high-risk patients exhibit higher activated DC scores.

The TMB denotes the mutation frequency per megabase of DNA. A high TMB can result in the presentation of additional novel antigens, which enhances immune system recognition and targeting of the tumor. This increased antigenic diversity may make the tumor more responsive to immunotherapy drugs, such as immune checkpoint inhibitors [31,32,33]. In this study, it was observed that high-risk patients exhibited higher TMBs. However, when considering the predictive results of immunotherapy, these high-risk patients showed a greater risk of immune evasion, which contradicts the aforementioned theory. Based on immune infiltration analysis, we speculate that this is due to the immunosuppressive tumor microenvironment observed in high-risk patients. Many studies have reported that TMB alone is not a definitive predictor of immunotherapy efficacy and should be considered alongside multiple clinical indicators [34,35]. Genomic instability can cause rapid mutations in tumor cells, constantly altering the antigenic landscape and hindering effective immune recognition and attack [36,37]. This could be why patients with a high TMB experience poor outcomes with immunotherapy.

JQ1 is a type of BET protein inhibitor. BET proteins modulate gene transcription by attaching to acetylated histone marks on chromatin. JQ1 mainly binds to the bromodomain of BET proteins, preventing their interaction with acetylated histones, thereby inhibiting the initiation and elongation of transcription [38,39]. Shi et al. [40] reported that JQ1 can alter cytokine expression in T cells and dendritic cells, which may have significant clinical implications for oncology treatment. In our study, a strong correlation between the sensitivity of JQ1 and the expression of five activated DC genes was observed. Molecular docking results further revealed that JQ1 had intimate binding with the proteins encoded by these five activated DCs genes, with binding energies all below −5 kcal/mol. We speculate that *PLCB2*, *XCR1*, *IFNG*, *HLA-DQB2*, and *SMIM24* may serve as potential competitive binding targets of JQ1, thereby participating in the activation process of DCs.

In the prognostic model, apart from the unclear function of the *SMIM24* gene, the other four genes, *PLCB2*, *XCR1*, *IFNG*, and *HLA-DQB2*, are all associated with immune regulation. *PLCB2*, a phospholipase C family enzyme, is crucial for cell signaling, particularly in the signaling pathway of G protein-coupled receptors (GPCRs). Upon GPCR activation, they generate secondary signals such as inositol trisphosphate (IP3) and diacylglycerol (DAG) through the activation of *PLCB2*. This process regulates intracellular calcium ion (Ca^2+^) concentration and activates protein kinase C (PKC) [41,42]. *PLCB2* also participates in regulating T-cell activation, differentiation, and immune response modulation [43]. The *XCR1*-encoded protein is a chemokine receptor that can bind to its ligands XCL1 and XCL2, thereby regulating the function of immune cells [43]. *XCR1* is crucial for immune regulation, particularly in modulating the activation and function of DCs. By binding to its ligands, *XCR1* regulates the localization, activation, and antigen presentation process of DCs, influencing the activation of immune cells and adaptive immune responses [44,45,46]. *IFNG* is a cytokine within the interferon protein family, produced by activated T lymphocytes, NK cells, and other immune cells. *IFNG* is crucial for immune regulation, as it boosts the antigen-presenting capabilities of macrophages and dendritic cells. Additionally, *IFNG* promotes T cell activation and proliferation, increases antibody production and cellular cytotoxic responses, and modulates the activity of macrophages and NK cells [46]. *HLA-DQB2* is a gene within the Human Leukocyte Antigen (HLA) system, encoding the *HLA-DQB2* molecule, which is an HLA class II molecule. It is also crucial for immune regulation. Together with other HLA class II molecules, it participates in the antigen presentation process by binding to exogenous antigens and presenting them to CD4^+^ T cells, thereby triggering specific immune responses. HLA class II molecules are also involved in regulating interactions between immune cells, including coordinating the actions of T and B cell activities and sustaining immune tolerance [47,48].

However, this study has certain limitations. Firstly, although our study has been validated across three independent cohorts, further validation in large-scale prospective cohorts is still required. In addition, more clinical factors should be included in the construction of the nomogram, such as smoking duration, obesity, and the presence of hypertension, as these are known risk factors for ccRCC. Including them could enhance the accuracy of the predictive model, thereby providing greater value for clinical decision-making. Unfortunately, the clinical data collected in the TCGA-KIRC cohort are incomplete. Incorporating too many clinical factors could reduce the total sample size available for constructing the nomogram, which might affect its stability and reliability. Although the expression of the five activated DC genes has been validated in 15 tissue samples, a larger sample size is necessary. Lastly, this study aims to identify prognostic markers and potential therapeutic targets for ccRCC using bioinformatics approaches, and the exploration of the specific biological mechanisms of the five activated DC genes is limited.

## 4. Materials and Methods

### 4.1. Calculation and Collection of ccRCC Tissue Samples

In this study, based on the expression data from the TCGA-KIRC cohort, the effect sizes of SMIM24, PLCB2, HLA-DQB2, IFNG, and XCR1 were calculated as −0.8597376, 2.053932, 1.786839, 1.400324, and 0.7872571, respectively. Using the pwr R package (version 1.30) with a power of 0.80 and an α error of 0.05, the required sample sizes were determined to be 13, 5, 5, 7, and 15 pairs of samples, respectively. In conclusion, we decided to collect 15 pairs of tissue samples. From March to August 2022, 15 pairs of surgically resected ccRCC tissues and corresponding adjacent normal tissues were obtained from the Department of Urology at the First Affiliated Hospital of Chongqing Medical University. Details of the patients’ clinical information can be found in Table S3. The collection process was supervised by the ethics committee and approved by the Biomedical Ethics Committee of Chongqing Medical University (Approval No: 2024018). Informed consent was obtained from all patients prior to surgery, and post-operative pathological reports and H&E staining confirmed the samples as ccRCC. Immediately after surgical resection, the samples were sliced to the size of a green bean. They were washed with 10 mL of PBS for five minutes to thoroughly remove all blood or contaminants on the surface. After washing, the PBS was completely removed, and the samples were placed in RNAlater solution (Qiagen, Hilden, Germany). The tissue samples were initially stored at −4 °C overnight and then transferred to −80 °C for long-term storage.

### 4.2. Raw Data

The training set of ccRCC was obtained from the TCGA-KIRC cohort, which includes RNA-seq, somatic mutation data, and clinical data, consisting of 531 ccRCC samples and 72 normal kidney tissue samples. Patients with OS of <30 days or missing survival status information were excluded, leaving 525 ccRCC samples for subsequent analyses.

Microarray data and clinical information for the validation set of ccRCC were obtained from the GEO database, ArrayExpress dataset, and ICGC database. The GSE167573 cohort includes 55 ccRCC samples, the GSE53757 cohort includes 72 pairs of matched ccRCC and adjacent non-tumor tissues, the E-MTAB-1980 cohort includes 101 ccRCC samples, and the ICGC cohort includes 233 ccRCC samples.

The single-cell dataset GSE152938 includes one normal kidney sample, two ccRCC samples, one chromophobe renal-cell carcinoma sample, and one papillary renal-cell carcinoma sample.

### 4.3. Immune Cell Infiltration Analysis

The ssGSEA algorithm (version 1.48.3) was employed to calculate the enrichment scores of 28 immune gene sets, quantifying the immune cell function [49]. The gene sets for 28 immune cells were downloaded from the TISIDB database, comprising a total of 782 genes (Table S4). Additionally, a correlation analysis was conducted between the immune cell infiltration calculated by the ssGSEA algorithms and the risk scores in the TCGA-KIRC cohort.

### 4.4. Differential Analysis

The DESeq2 package (version 1.40.2) was utilized to identify 2318 DEGs between ccRCC and normal kidney tissue in the TCGA-KIRC cohort, with |log_2_ (fold change)| > 1 and adjusted *p* < 0.05.

### 4.5. Weighted Gene Co-Expression Network Analysis (WGCNA)

WGCNA (version 1.72.1) aims to discover and identify co-expression patterns of gene modules related to specific biological processes, diseases, or other phenotypes. The co-expression network was constructed using 2318 DEGs and the activated DC scores. A power scatter plot was created, and the optimal soft threshold (β = 4) was selected to obtain the gene pairwise correlation matrix. Then, genes were clustered and modules were dynamically identified and cut (minModules Size = 80). The patterns of gene modules were plotted, and similar modules were merged. The minimum number of module genes was set to 80 and a correlation test was performed to examine the correlation between activated DC scores and modules. A heatmap was then generated. Finally, the two modules most relevant to the activated DC subtypes were extracted for further analyses.

### 4.6. Preprocessing of Single-Cell Data

GSE152938 was first transformed into a Seurat object. Cells failing quality control, defined as having gene counts < 200 or >5000, or mitochondrial gene percentages > 10%, were excluded. A total of 12,915 cells passed quality control and were used for subsequent analysis. The “FindVariableFeatures” function identified the top 2000 highly variable genes. These genes were then normalized and subjected to principal component analysis using the “NormalizeData” function with the “LogNormalize” method. Subsequently, the “RunTSNE” and “FindClusters” functions clustered cells at a resolution of 0.7. The “FindAllMarkers” function identified marker genes for each cluster. Cell annotation was performed using the CellMarker database and markers from the dataset’s original literature [50].

### 4.7. Identification of Activated DC Genes at the Single-Cell Transcriptomic Level

To explore the activation status of DCs, the enrichment scores of the activated DC-related gene set from the TISIDB database were investigated using the “AddModuleScore” function. The AddModuleScores function calculates the relative expression of a gene set within each cell, incorporating a control from a background gene set, to provide a score that reflects the activity level of certain biological processes or pathways within the cell. Based on the median enrichment scores, cells were divided into activated DCs and non-activated DCs. The “FindMarkers” function identified marker genes for activated DCs with log_2_ (fold change) > 1 and *p* < 0.05 (Wilcoxon test). At the single-cell transcriptomic level, the marker genes were considered as the activated DC genes.

### 4.8. Construction of Activated DC Prognostic Modules

Firstly, univariate Cox regression analysis was employed to preliminarily identify genes linked to the prognosis, with *p* < 0.05. Subsequently, LASSO regression analysis was conducted, and lambda.1se was selected to reduce data dimensionality and eliminate redundant genes. Finally, multivariable Cox regression analysis was conducted to identify genes with *p* < 0.01 to construct prognostic models. The risk scores for ccRCC patients were calculated based on the coefficients from the multivariable Cox regression analysis and the corresponding gene expression. The median risk score served as the cutoff to divide ccRCC patients into two groups. Using the “survival” R package, KM survival curves were plotted to identify prognostic differences between the two groups. Furthermore, the “timeROC” R package was utilized to plot ROC curves.

### 4.9. Drug Sensitivity

The “OncoPredict” R package (version 0.2) utilizes machine learning models, combining gene expression data with existing drug response data, to predict tumor patients’ sensitivity to different chemotherapy drugs. In this study, the IC50 of common chemotherapy and targeted drugs for ccRCC patients in the TCGA-KIRC cohort was estimated.

### 4.10. Immunotherapy Prediction

The TIDE score predicts patient response to immune checkpoint inhibitors, primarily targeting PD-1, PD-L1, and CTLA-4, and is used to assess immune evasion in response to immunotherapy. The TIDE scores calculated in this study are based on bulk transcriptome data from the TCGA-KIRC cohort.

### 4.11. Tumor Mutation Analysis

To quantify the tumor mutation landscape, the R package “maftools” and R (version 3.4) were used to plot waterfall plots and calculate the TMB score for each ccRCC patient in the TCGA-KIRC cohort.

### 4.12. Molecular Docking

The potential binding sites of five activated DC genes with JQ1 were predicted through molecular docking. The conformation of JQ1 was downloaded from the PubChem database. Protein structures of IFNG and PLCB2 were retrieved from the PDB database with accession numbers 1eku and 2fju. The protein structures of HLA-DQB2, SMIM24, and XCR1 were downloaded from the AlphaFold database.

AutoDock (version 1.5) was employed to dock molecules of JQ-1 with the core targets of five proteins, and a genetic algorithm was employed to search for the docking conformations.

### 4.13. qRT-PCR

RNA extraction from ccRCC tissues and adjacent tissues was performed using the RNaiso PLUS Kit (Takara, Dalian, China). RNA was converted to cDNA using the Eastep@ RT Master Mix Kit (Promega, Beijing, China) for subsequent analysis. The qRT-PCR reaction mixture consisted of 2 × Universal SYBR Green Fast qPCR Mix (5 μL), DEPC-treated water (3.2 μL), forward/reverse primers (0.3 μL each), and cDNA (1.2 μL). mRNA expression was calculated by 2^−ΔΔCt^ normalized to *GAPDH*. See Table 2 for qRT-PCR primers.

## 5. Conclusions

In summary, we established a robust prognostic model for activated DCs by integrating single-cell and transcriptomic data. This model has been validated across three independent cohorts, allowing for the prediction of prognosis in ccRCC patients and their responses to immune and targeted therapies.

## Figures and Tables

**Table 1:** The predictive binding energy between target proteins and JQ-1.

Gene	Drug	Predicted Binding Energy (Kcal/mol)
*SMIM24*	JQ-1	−5.1
*IFNG*	JQ-1	−7.4
*HLA-DQB2*	JQ-1	−6.2
*XCR1*	JQ-1	−6.9
*PLCB2*	JQ-1	−7.7

**Table 2:** qRT-PCR primers.

Gene	Forward Primers	Reverse Primers
*PLCB2*	5′-ATGGAGTTCCTGGATGTCACG-3′	5′-CGGAGTTTCTGGCTCTTGGG-3′
*XCR1*	5′-CCTACGTGAAACTCTAGCACTGG-3′	5′-AAGGCTGTAGAGGACTCCATCTG-3′
*IFNG*	5′-GGAGGAACTGGCAAAAGGAT-3′	5′-TTCAAGACTTCAAAGAGTCTGAGG-3′
*HLA-DQB2*	5′-GTGTGCAGACACAACTACGAGG-3′	5′-TCACTGAGCAGACCAGCAGGTT-3′
*SMIM24*	5′-AAGGAAGGAGAGAGCAACTTGG-3′	5′-CACATGACTGTGCTCTTTGCTC-3′
*GAPDH*	5′-GGTGTGAACCATGAGAAGTATGA-3′	5′-GAGTCCTTCCACGATACCAAAG-3′

## Data Availability

The original data were sourced from the TCGA, GEO, ICGC, and ArrayExpress databases. Detailed information regarding data download sources and processing methods is provided in the Methods section of this manuscript. Data generated during this work can be obtained by contacting the corresponding author.

## Supplementary Materials

The following supporting information can be downloaded at: https://www.mdpi.com/article/10.3390/ijms25179235/s1.
