# Supplementary material for: Integrating Bulk and Single-Cell RNA-Seq Data to Identify Prognostic Features Related to Activated Dendritic Cells in Clear-Cell Renal-Cell Carcinoma
Source: Int J Mol Sci. 2024 Aug 26;25(17):9235. doi: 10.3390/ijms25179235 (PMC11395106; doi:10.3390/ijms25179235)
Supplement: Supplementary file 1 [file ijms-25-09235-s001.zip › Supplementary Figure.pdf]

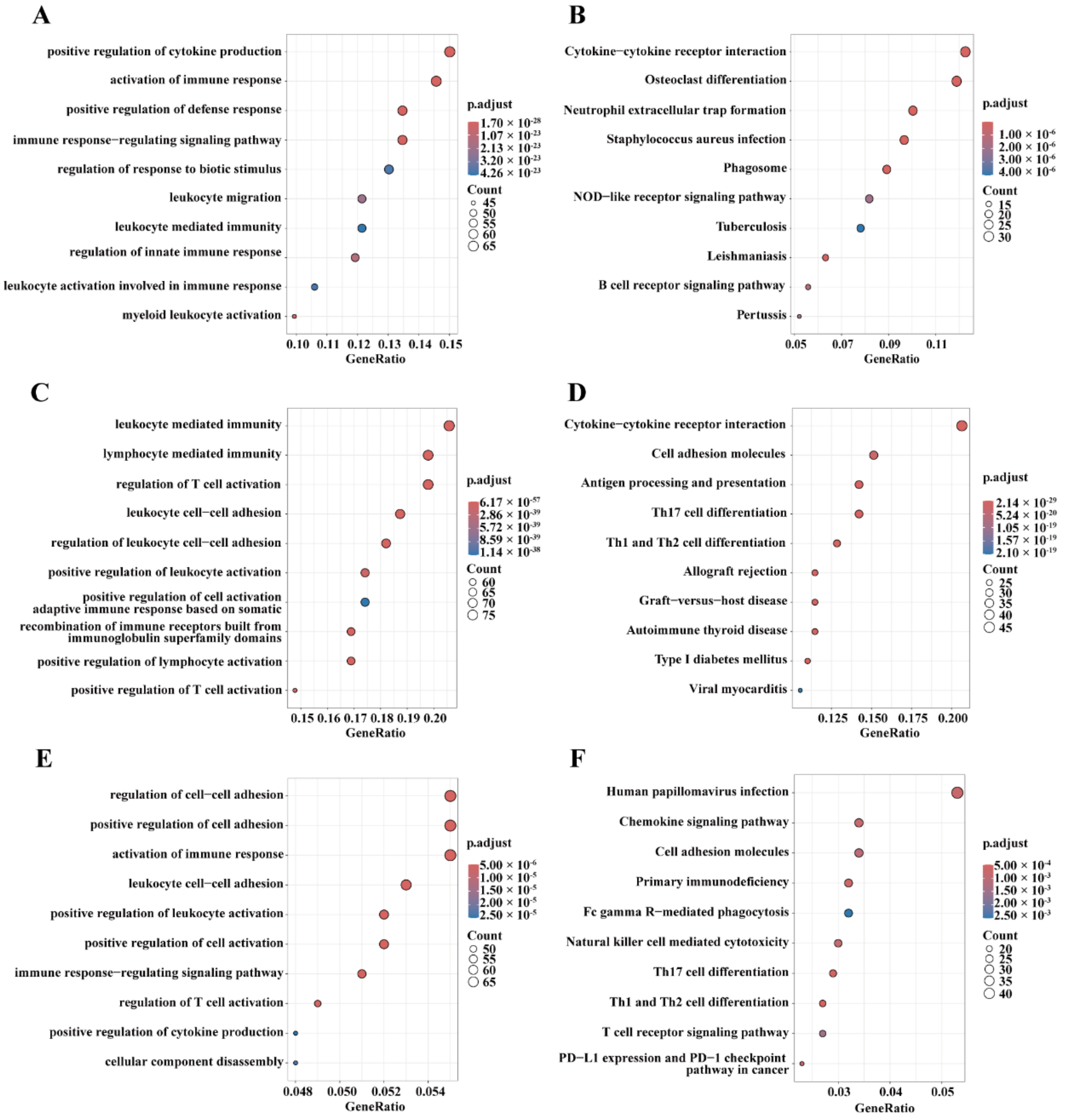

Supplementary Figure 1. Enrichment analysis. The GO enrichment results and KEGG enrichment results of (A, B) brown module, (C, D) red module, and (E, F) activated DCs genes identified in the single-cell transcriptome.

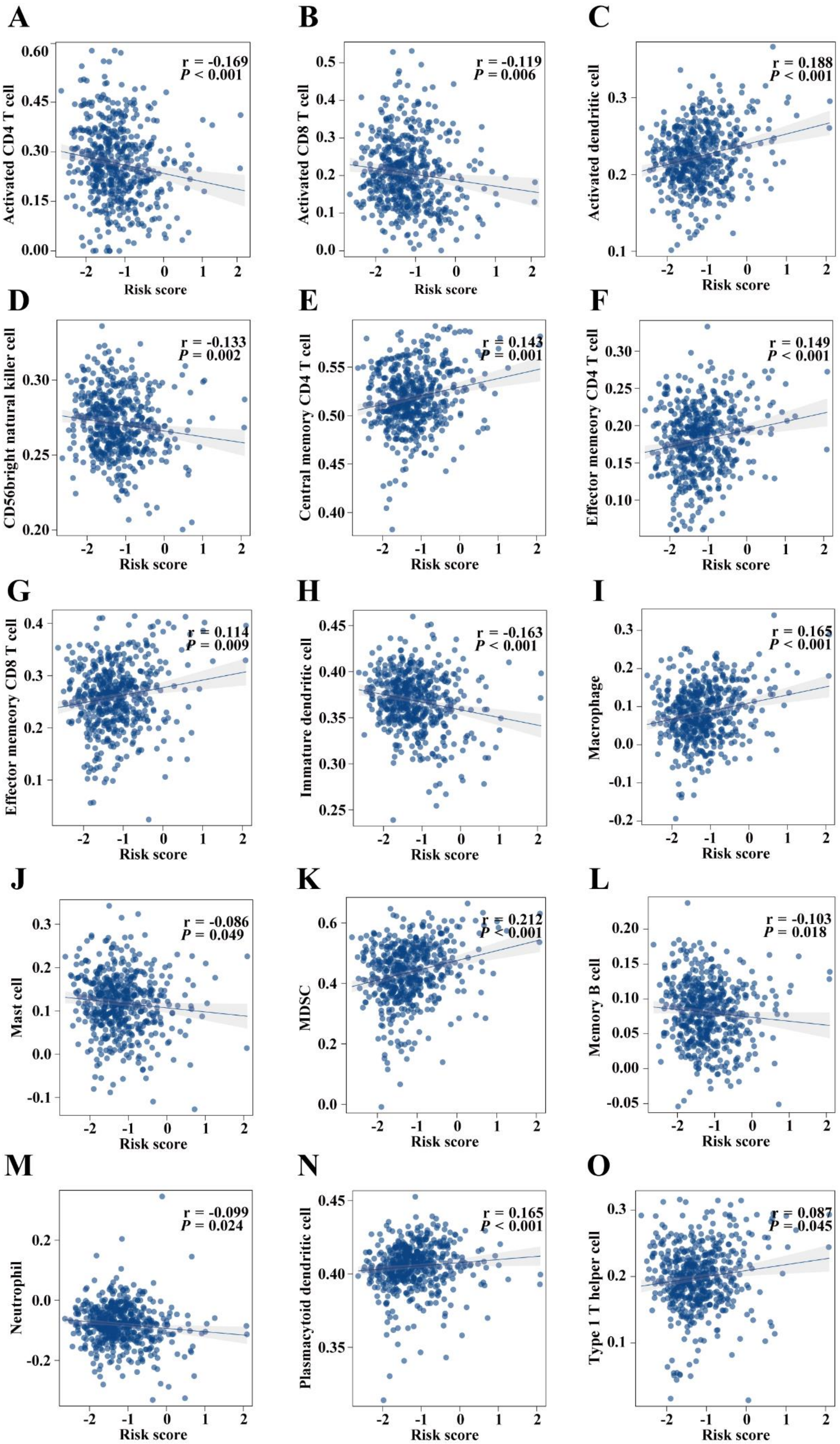
A
r = -0.169
P < 0.001
Activated CD4 T cell
Risk score
B
r = -0.119
P = 0.006
Activated CD8 T cell
Risk score
C
r = 0.188
P < 0.001
Activated dendritic cell
Risk score
D
r = -0.133
P = 0.002
CD56bright natural killer cell
Risk score
E
r = 0.143
P = 0.001
Central memory CD4 T cell
Risk score
F
r = 0.149
P < 0.001
Effector memeory CD4 T cell
Risk score
G
r = 0.114
P = 0.009
Effector memeory CD8 T cell
Risk score
H
r = -0.163
P < 0.001
Immature dendritic cell
Risk score
I
r = 0.165
P < 0.001
Macrophage
Risk score
J
r = -0.086
P = 0.049
Mast cell
Risk score
K
r = 0.212
P < 0.001
MDSC
Risk score
L
r = -0.103
P = 0.018
Memory B cell
Risk score
M
r = -0.099
P = 0.024
Neutrophil
Risk score
N
r = 0.165
P < 0.001
Plasmacytoid dendritic cell
Risk score
O
r = 0.087
P = 0.045
Type 1 T helper cell
Risk score

Supplementary Figure 2. The correlation analysis between immune cells in ssGSEA and risk score. Correlation analysis between the scores of (A) Activated $CD4^+$ T cell, (B) Activated $CD8^+$ T cell, (C) Activated dendritic cell, (D) CD56bright natural killer cell, (E) Central memory $CD4^+$ T cell, (F) Effector memory $CD4^+$ T cell, (G) Effector memory $CD8^+$ T cell, (H) Immature dendritic cell, (I) Macrophage, (J) Mast cell, (K) MDSC, (L) Memory B cell, (M) Neutrophil, (N) Plasmacytoid dendritic cell, (O) Type 1 T helper cell, and the risk score.
